# Supplementary material for: ChatGPT as a Tool for Biostatisticians: A Tutorial on Applications, Opportunities, and Limitations
Source: Stat Med. 2025 Oct 23;44(23-24):e70263. doi: 10.1002/sim.70263 (PMC12548020; doi:10.1002/sim.70263)
Supplement: Supplementary file 1 — Data S1. Supporting Information. [file SIM-44-0-s001.zip › Supplementary Materials Additional.zip!/3.1_Systematic_Reviews_and_Meta_aspects/3.1_Data Extraction - o4-mini - Chat 9.pdf]

Use Case Meta-Analysis - Data Extraction - o4-mini - Chat 9

Rudloff et al. - 2009 - Concurrent lobular neoplasia increases the risk of ipsilateral breast cancer recurrence in situ treated with breast-conserving therapy.pdf
PDF

Vidali et al. - 2012 - Conservative treatment of breast ductal carcinoma in situ results of an Italian multi-institutional.pdf
PDF

The aim is to perform a meta-analysis on total ipsilateral local recurrence rates at 10 years in cases of ductal carcinoma in situ.

Data inclusion rules are: To ensure the validity of the follow-up data, the results should be as close to 10 years follow-up (minimum) as the information in each study allows. If there is more than one publication from an institution or group, the most recent study with appropriate follow-up will be used to extract 10-year data.

Please provide from each of the two attached studies:

- the rate of ipsilateral local recurrence for conservative surgery with additional radiotherapy (no other interventions or drugs allowed) after 10 years

- the number of eligible patients

- period of data collection

- duration of follow-up

- type of study

- age of patients (mean or median, maximum and minimum)

- treatment modalities

For this task you have access to up-to-the-minute web retrieval to find adequate approaches for data extraction. When answering the task above please proceed as follows:

1. Fetch the most relevant sources.

2. Show your step-by-step reasoning.

3. At the end, give me a concise answer citing the sources you used.

4. Please provide a dataframe with one entry for each study, and column names "study", "rate", "n_patients", "period", "follow_up", "study_type", "age", and "treatment".

Meta-analysis Dataset

	study	rate	n_patients	period
1	Rudloff et al. (2009)	11.9%	91	1991–1995
2	Vidali et al. (2012)	9.6%	586	February 1985–March 2000

Step-by-step reasoning:

1. **Identify studies**
    - *Rudloff et al.* (2009) is a single-institution, retrospective cohort of 294 DCIS patients treated with breast-conserving therapy between 1991–1995, of whom 91 received external beam radiotherapy (no chemotherapy; 6.1% received tamoxifen).
    - *Vidali et al.* (2012) is a multi-institutional, retrospective Italian study of 586 DCIS patients treated with conservative surgery + whole-breast RT (50 Gy median dose; boost in 50%) between February 1985–March 2000.
2. **Extract 10-year ipsilateral recurrence rates**
    - *Rudloff et al.* report a 10-year local recurrence rate of **11.9%** for women receiving surgery + radiation versus 27.9% without radiation.
    - *Vidali et al.* show an actuarial risk of local recurrence of **9.6%** at 10 years.
3. **Gather other required data**
    - **Number of eligible patients:** 91 in *Rudloff et al.* (those receiving RT) and 586 in *Vidali et al.*
    - **Period of data collection:** 1991–1995 (*Rudloff*); February 1985–March 2000 (*Vidali*).
    - **Duration of follow-up:** Median 11 years (range 0–16) for *Rudloff*; median 136 months (range 16–292) for *Vidali*.
    - **Type of study:** Retrospective institutional cohort (*Rudloff*) and multi-institutional retrospective (*Vidali*).

- **Age of patients:** Median 55 years (range 26–89) in *Rudloff*; median 55 years (range 29–84) in *Vidali*.
- **Treatment modalities:** Breast-conserving surgery + external beam RT (no chemo; tamoxifen in 6.1%) in *Rudloff*; conservative surgery + whole breast RT (median 50 Gy; boost in 50%) in *Vidali*.

Below is a consolidated dataset for each study:

python_user_visible

Quellen
